# Contributions of NMR to the Understanding of the Coordination Chemistry and DNA Interactions of Metallo-Bleomycins

**DOI:** 10.3390/molecules18089253

**Published:** 2013-08-02

**Authors:** Teresa Lehmann, Elena Topchiy

**Affiliations:** Department of Chemistry, University of Wyoming, Laramie, WY 82071, USA; E-Mail: etopchiy@uwyo.edu

**Keywords:** antibiotic, cancer, bleomycin, solution structures

## Abstract

Bleomycins are a family of glycopeptide antibiotics that have the ability to bind and degrade DNA when bound to key metal ions, which is believed to be responsible for their antitumor activity. Knowledge of the structures of metallo-bleomycins is vital to further characterize their mechanism of action. To this end, numerous structural studies on metallo-bleomycins have been conducted. NMR spectroscopy has had a key role in most of these studies, and has led to very important findings involving the coordination chemistry of metallo-bleomycins, and the details of many metallo-bleomycin-DNA spatial correlations for this important drug. This paper reviews the most important contributions of NMR to the bleomycin field.

## 1. Introduction

**Figure 1:**
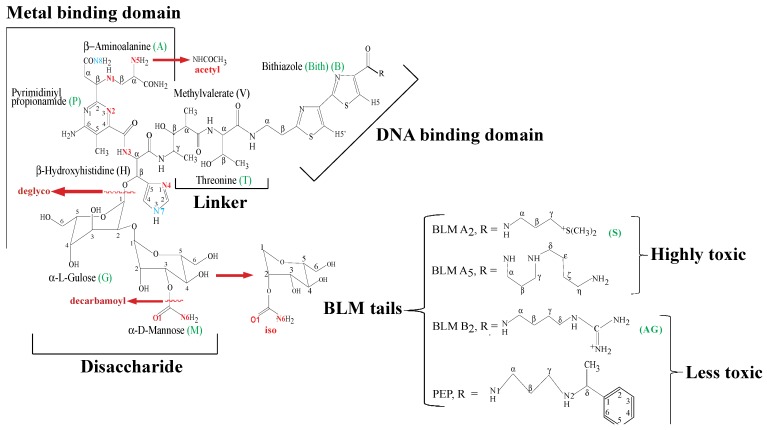
Structures of BLM-A_2_, -A_5_, -B_2_, and PEP. The coordinating atoms postulated by most researchers are labeled and colored in red. Other coordination sites also proposed in the literature are labeled and colored in blue. BLM residue abbreviations are colored in green.

The antitumor antibiotics known as bleomycins (BLMs) are complex glycopeptides isolated from cultures of *Streptomyces verticillus* (Figure 1). BLMs are DNA-cleaving compounds used as cancer chemotherapy agents in a mixture called blenoxane, consisting mainly of BLM-A_2_ and -B_2_ [1,2,3,4]. The drug acts as an antitumor agent by virtue of the ability of a metal complex of the antibiotic to cleave DNA [5,6,7,8]. The difficulties presented by the crystallization of metallo-BLMs (MBLMs) prompted their structural characterization by means of NMR. Improvements of this spectroscopic technique led in turn to further expansion of the knowledge base for this important anticancer drug. NMR has provided key information regarding the coordination chemistry and DNA interactions of MBLMs. The crystal structure of Cu(II)-P3A, a biosynthetic intermediate of BLM, became available soon after the discovery of BLM [9]. However, this peptide is missing the DNA-binding domain and the disaccharide fragments present in the full drug, and only provided limited information about its metal complexes. The first crystal structures of fully-formed BLMs were reported by Sugiyama *et al*. [10] in 2002 when BLM and Cu(II)BLM were crystalized in a complex with a BLM resistance determinant from BLM-producing *S. verticillus*. This crystal structure was followed by that of DNA-bound Co(III)BLM-B_2_ reported by Goodwin *et al*. [11]. In general, all of these structures confirmed many findings regarding the total structure of BLM, the coordination chemistry, and DNA interactions of MBLM previously assessed through NMR. This paper reviews and updates the most important contributions of NMR to the BLM field.

## 2. *Apo-*BLMs

Among the first NMR studies on BLM are those performed by Takita *et al.* [12] and Naganawa *et al.* [13] These studies involved a collection of ^13^C-NMR spectra of amino acid components, terminal amines, and sugar and peptide fragments of BLM; as well as BLMs with minor modifications. The chemical shift information gathered in this research effort, together with natural abundance ^15^N-NMR spectra collected for BLM-A_2_ [14], set up the basis for the determination of the total structure of BLM. BLM-A_2_ and -B_2_ are the two main components of the isolates from *Streptomyces verticillus* found by Umezawa *et al.* [15]. NMR work performed on these two congeners by Chen *et al.* [16] indicated that their ^1^H-NMR signals display nearly identical chemical shifts, with the exception of those generated by the C-termini substituents (Figure 1). These findings allowed the study of paramagnetic MBLMs such as Fe(II)BLM, using the clinical mixture of BLMs, blenoxane, without previous separation of BLM-A_2_ and -B_2_ (*vide infra*).

The aforementioned studies did not provide the complete assignments of the ^1^H-NMR spectrum of BLM due to many overlapping signals. Additionally, some of the resonances of the exchangeable protons in this molecule were also unassigned. The development in NMR spectroscopy occurred between 1979s and 1984s extended the applicability of this technique, and led to the full assignment of the proton signals of BLM-A_2_ by means of two-dimensional techniques [17]. Two-dimensional NMR spectroscopy was also used to fully assign the ^1^H-NMR spectra of the BLM congener peplomycin (PEP) [18,19] (Figure 1) at 900 MHz resolution [20]. The NMR data generated in this study was used to model the solution structure of PEP. An extended structure was found based on the lack of non-trivial NOEs for this compound. The NMR studies described above set up the basis for the study of MBLMs in solution.

## 3. Metallo-BLMs

### 3.1. Zn(II)BLM Complexes

Early after the discovery of BLMs, it was established that a metal ion cofactor was required by the drug for its *in vivo* as well as *in vitro* activity [5]. This discovery prompted the investigation of the coordination chemistry of MBLMs. Amongst the first reports in this area are those of Dabrowiak *et al*. [21] and Cass *et al*. [22] to define the nature of the interactions between BLM-A_2_ and Zn(II). The chemical shift changes exhibited by the signals generated by the imidazole and pyrimidine rings in BLM upon addition of Zn(II) was used by these authors to propose their coordination to the Zn(II) ion. Other possible coordination sites could not be confirmed by NMR at this time, due to the intense overlap of the ^1^H signals in the one-dimensional spectra collected for this MBLM. Attempts to study the coordination chemistry of Cu(II)BLM through ^1^H-NMR were made by Dabrowiak at this time [23]. However, the extreme paramagnetic nature of the Cu(II) center prevented the identification of its metal ligands in this study. On the basis of the ^13^C assignments for BLM provided by Naganawa *et al.* [13], Dabrowiak and co-workers collected ^13^C-NMR spectra of Zn(II)BLM-A_2_. The use of ^13^C-NMR in diamagnetic Zn(II)BLM confirmed the coordination of the Zn ion to the imidazole and pyrimidine rings, and hinted the binding of the carbamoyl-NH_2_ nitrogen in the mannose moiety (M) and the primary amine in β-aminoalanine (A).

The kinetics of dissociation of the Zn(II)BLM complex was determined through NMR by Lenkinski and co-workers [24]. A 1:1 Zn(II):BLM stoichiometry was determined for the Zn(II)BLM complex, based on the relative intensity of the imidazole C4 proton signal at various Zn(II) concentration. A value of (3.0 ± 2.0) × 10^−2^ (s^−1^) was found for the rate of dissociation of Zn(II). Comparison of this value with those determined for carbonic anhydrase and carboxypeptidase, where the Zn ion is in a distorted tetrahedral environment, led these researchers to conclude that Zn(II) is also in a tetrahedral environment in Zn(II)BLM. ^1^H-NMR was also used to determine the tertiary structure of Zn(II)BLM and the conformational changes associated with metal binding to BLM [25]. The largest changes in chemical shift occurred for the protons in the pyrimidinylpropionamide (P), A, β-hydroxyhistidine (H), and M moieties of BLM upon Zn(II) binding. These results were used to propose the coordination of the Zn ion to P ring, A primary and secondary amines, H imidazole, and M carbamoyl group. The methylvalerate (V) moiety also displayed large changes in chemical shift upon Zn(II) complexation. These changes were attributed to the location of V above the imidazole ring in the Zn(II)BLM complex (ring current effects). However, the possibility of coordination of the V carbonyl oxygen to the metal center was not ruled out in this study. NMR spectroscopy was also used to study the coordination chemistry of Zn(II)(Acetyl-BLM) (Figure 1) [26]. Acetyl-BLM is an analog of BLM that does not degrade DNA, where the α-amino group of the A moiety has been N-acetylated. The results of this study indicated that Zn(II)-bound BLM and Acetyl-BLM share the P ring, H imidazole, M carbamoyl, and A secondary amine as ligands to the metal center. It was proposed that the acetylation of the A α-amino group prevents the coordination of the A secondary amine to the metal center, and produces an inactive metal complex unable to cleave DNA in the presence of iron.

**Figure 2:**
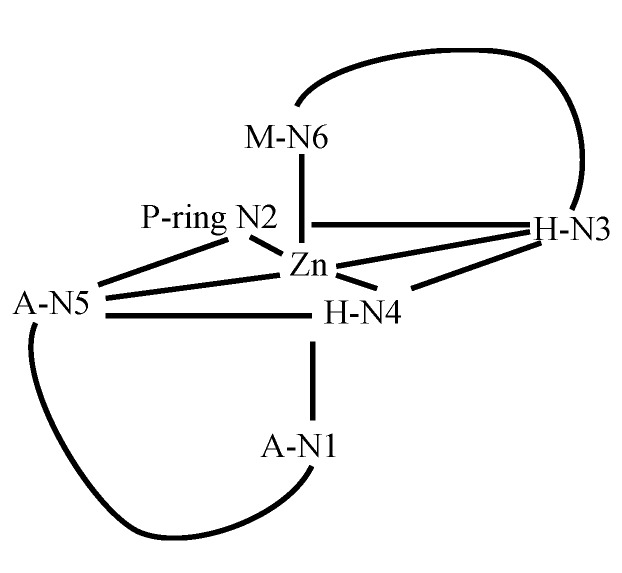
Schematic drawing of the Zn(II)-coordination site in Zn(II)BLM proposed by Akkerman *et al.* [27].

The complete assignment of the ^1^H- and ^13^C-NMR signals for Zn(II)BLM was only achieved through the use of two-dimensional NMR spectroscopy [27,28,29]. Comparison of the *apo-* and Zn(II)-bound BLM spectra indicated that, as established previously by other research groups (*vide supra*), the A, P, H, and M residues in BLM showed the most dramatic chemical shift changes upon metal coordination [27,28]. The observation that mostly *trans* rotamers were detected in the NMR spectra of Zn(II)BLM, indicated that the α and β protons of the H residue were in a more or less fixed conformation. These results were used to propose the coordination of the H amide nitrogen to the Zn ion, which was also supported by the deprotonation of the amide proton of fragment H in the presence of Zn(II). The coordination of the carbamoyl group of M was suggested, based on the observation of two broadened singlets in the spectrum of Zn(II)BL *vs*. only one broadened singlet in *apo-*BLM, and the ^1^H chemical shift change detected for the M-H3 proton upon Zn(II) complexation. NOE cross peaks connecting the V-threonine (T) fragment to the H and B moieties were used to propose that this fragment was oriented bringing the residue V close to the imidazole ring. This could explain the significant changes in chemical shifts observed for V in the Zn(II)BLM spectra when compared to those of *apo-*BLM. The NMR data derived from this investigation was used to calculate the first solution structure of a MBLM. The coordination symmetry around the Zn ion was proposed to be in between octahedral and trigonal prismatic. A schematic drawing of the Zn(II) coordination site proposed is shown in Figure 2.

The Zn(II) complex of tallysomycin (TLM), a BLM analog containing an additional sugar (4-amino-4,6-dideoxy-L-talose) and lacking a methyl group in the V moiety, was also examined through NMR spectroscopy [30]. Coordination of the A primary and secondary amines, P and H rings, and H amide nitrogen were also proposed in this study. Although chemical shift changes of the ^13^C and ^1^H resonances were observed upon Zn(II) complexation, they were not attributed to the coordination of the carbamoyl group to the Zn ion, but to changes in conformation and positioning of the disaccharide unit on complexation. The aforementioned studies allowed the identification of the ligands to the metal center in Zn(II)BLM. Most of these ligands were also proposed for Fe- and Co-bound BLMs, and would be later confirmed through the crystal structures of Cu(II)- and HOO-Co(III)BLM [10,11].

### 3.2. CoBLM Complexes

Initial interest in CoBLM complexes aroused when radioactive ^57^CoBLM complexes were used as radiopharmaceuticals in tumor-localizing studies [31,32,33]. Additionally, Co(II)BLM was proposed to be isostructural with Fe(II)BLM [34] CoBLMs were considered powerful spectroscopic probes since ESR, NMR and optical spectroscopies could all be applied to these complexes. NMR studies on Co(II)BLM performed by Bereman *et al.* [35] were unsuccessful due to the paramagnetic nature of this complex. However, oxidation of Co(II)BLM revealed a sharp set of NMR signals. Comparison of this spectrum with that of *apo-*BLM, showed changes in chemical shifts for protons in the imidazole and pyrimidine rings. This finding was used to suggest the coordination of the P and H moieties to the metal ion in oxidized Co(II)BLM.

**Figure 3:**
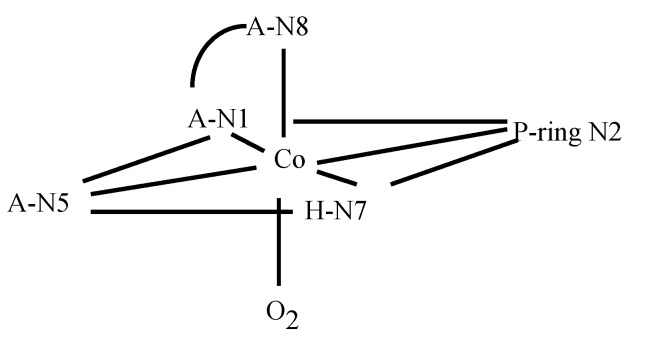
Coordination sphere of the Co(III) ion in Co(III)BLM proposed by Vos *et al.* [38].

Analysis of the oxidation products of Co(II)BLM by various scientists [36,37] led to the discovery of two BLM species denominated Form I (HOO-Co(III)BLM) and Form II (Co(III)BLM). Since their discovery, both MBLMs have been extensively studied. ^13^C-NMR spectroscopy was used to detect the changes in chemical shifts that take place in *apo*-BLM upon complexation with Co(II) [38]. The broadening of the signals in the ^13^C-NNR spectra of Co(II)BLM indicated that P, and A, as well as M were involved in metal binding. Surprisingly, no significant changes in ^13^C-signal shifts were detected for H, which led to its exclusion as a metal coordination point. Oxidation of Co(II)BLM was conducted in this study, and ^13^C-NMR spectra derived from Co(III)BLMs were examined, resulting in proposal of the coordination structure shown in Figure 3. The coordination scheme postulated in this study is different from the ones available at the time for Zn- and CuBLM (*vide supra*). The imidazole ring is postulated as a ligand through N7, and N3 is replaced by N8. Additionally N5 is proposed to be an equatorial coordination site together with N1.

**Figure 4:**
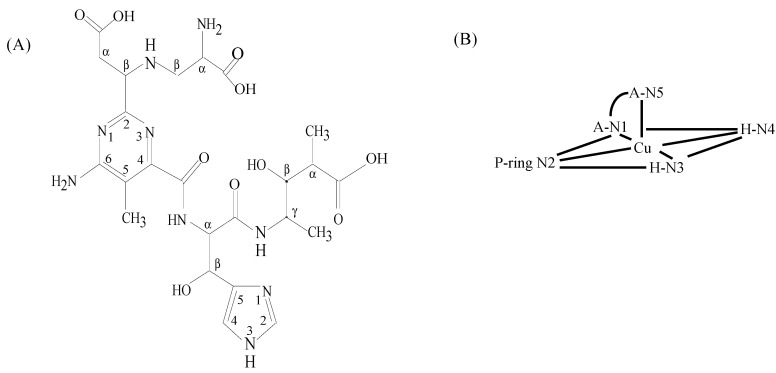
(**A**) Pseudotetrapeptide A and (**B**) Cu(II) coordination environment determined from the structure of Cu(II)-P3A [9].

The NMR investigation of the coordination chemistry of CoBLMs was facilitated by the study of the major hydrolysis product of CoBLM, pseudotetrapeptide A [39,40] (Figure 4A). This simplified molecule keeps most of the metal-ligation points of BLM, and produces simplified NMR spectra. A combination of ^1^H-NMR, absorption, and circular dichroism spectroscopies allowed the identification of the ligands to the metal center in this molecule, which coincided with those observed in the X-ray structure of Cu(II)(P-3A) (Figure 4B). This coordination structure was later confirmed by the crystal structure of DNA-bound Co(III)BLM-B_2_ reported by Goodwin *et al*. [11].

**Figure 5:**
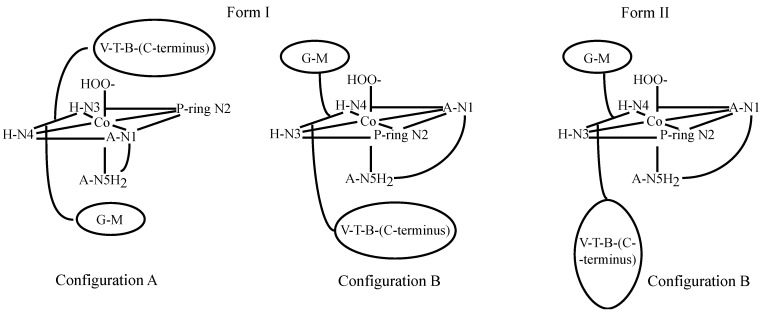
Conformations of Co(III)BLM Forms I and II proposed by Xu *et al*. [42].

The reaction of Co(II)BLM with O_2_ was investigated in detail [41]. It was determined that forms I and II of Co(III)BLM produced different NMR spectra, which indicated that the two species had different solution structures. NMR studies of both Forms I and II were carried out by Xu *et al*. [42]. NOESY spectra collected for both forms revealed that metal binding to N1, N3, N4, and N5 brings the A and H moieties together. Additionally, NOE correlations were found connecting H to V and T, indicating that metal complexation determined the conformation of the linker region. B to V, T, and P. NOESY connectivities revealed that the DNA-binding domain was in close proximity to the linker in both forms, and to the metal-binding domain in Form I. NMR-based MD calculations performed for both forms indicated that in Form I the B group is folded back upon the cobalt binding domain, while it has an extended conformation in Form II. Both forms also exhibited two different chiralities (configurations A and B) for the metal center, with the DNA-binding domain occupying different sides of the equatorial plane of the metal (Figure 5). Additionally, the MD calculations also indicated that there was preference for configuration A in Form I, and for configuration B in form II. Form I of Co(III)BLM is considered to be analogous to the activated form of FeBLM. This fact led to multiple research efforts to be concentrated on it.

**Figure 6:**
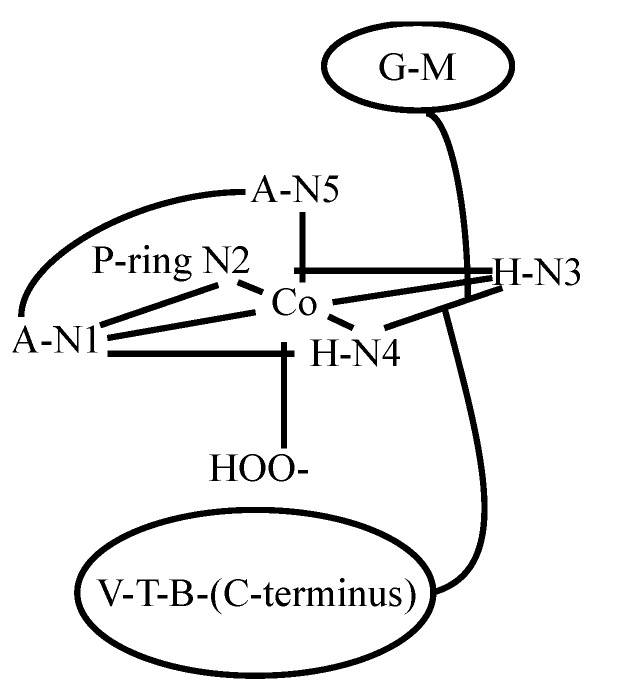
NMR-determined structure of HOO-Co(III)BLM-A2 [43].

A detailed NMR study of Co(III)BLM Form I was performed by Wu and co-workers [43]. The diamagnetic nature of this compound allowed the assignment of all ^1^H and ^13^C signals, as well as the determination of multiple *J*-coupling constants and non-trivial NOE connectivities. These data were used to model the solution structure of HOO-Co(III)BLM through molecular dynamics calculations. Examination of initial coordination models in light of the NMR data generated for HOO-Co(III)BLM led to the proposal of the structural conformation depicted in Figure 6. In this conformation, the primary amine in A is proposed as the endogenous axial ligand, and the linker and DNA-binding domain are folded back underneath the equatorial plane of the metal. This configuration of the V-T-B-(C-terminus) segment of BLM would be considered key for (MBLM)-DNA complex stabilization (*vide infra*).

**Figure 7:**
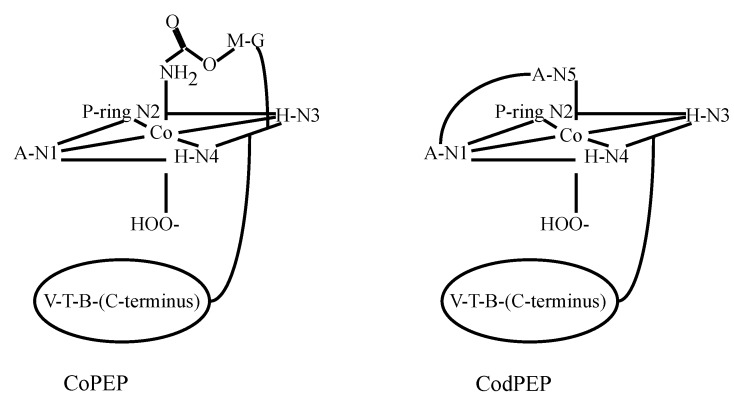
Coordination of scheme of Co(III) in HOO-CoPEP and HOO-CodPEP [47].

The BLM-family includes many members that differentiate from each other on the nature of the substituent located at the C-terminus of the drug (Figure 1). Among those members, PEP (Figure 1), which contains a 3-[(S)-1-phenyllethylamino]-propylamine as the C-terminal substituent, has been shown to exhibit higher antitumor activity and less pulmonary toxicity than BLM-A_2_ and -B_2_. [44,45,46]. These facts generated wide scientific interest in PEP. Previous NMR studies performed in Zn(II)- and CO-Fe(II)-bound BLM (*vide supra*) had suggested the coordination of the carbamoyl group of M to these metal centers as an axial ligand. However, the NMR investigations of HOO-Co(III)BLM referred to above postulated the primary amine in A to take the role of the endogenous axial ligand. NMR studies of HOO-Co(III)PEP (CoPEP) and its deglycosylated analog (CodPEP) were carried out in an attempt to diffuse the controversy involving nitrogens N5 and N6 [47] (Figure 1). The NMR data generated for both HOO-Co(III) congeners was used in molecular dynamics calculations and led to the structures shown in Figure 7. As can be seen from this figure, the primary amine in A is proposed as a ligand in CodPEP but not in CoPEP. The authors based these conclusions on the differences in chemicals shifts observed for the M-NH_2_ and A-NH_2_ protons in both Co(III) compounds. Large upfield shifts were observed for the A-NH_2_ protons in CodPEP (3.95/4.09 ppm), but not in CoPEP (5.93/6.58 ppm). Additionally, the exchange rate of the A-NH_2_ protons with water was found to be slow in CodPEP and fast in CoPEP, while it was determined to be fast for the M-NH_2_ protons in CoPEP. These results were used to propose that the primary amine in A only binds to the Co(III) in the absence of the disaccharide. In the presence of the G-M segment, the mannose-carbamoyl nitrogen becomes the preferred endogenous-axial ligand.

**Figure 8:**
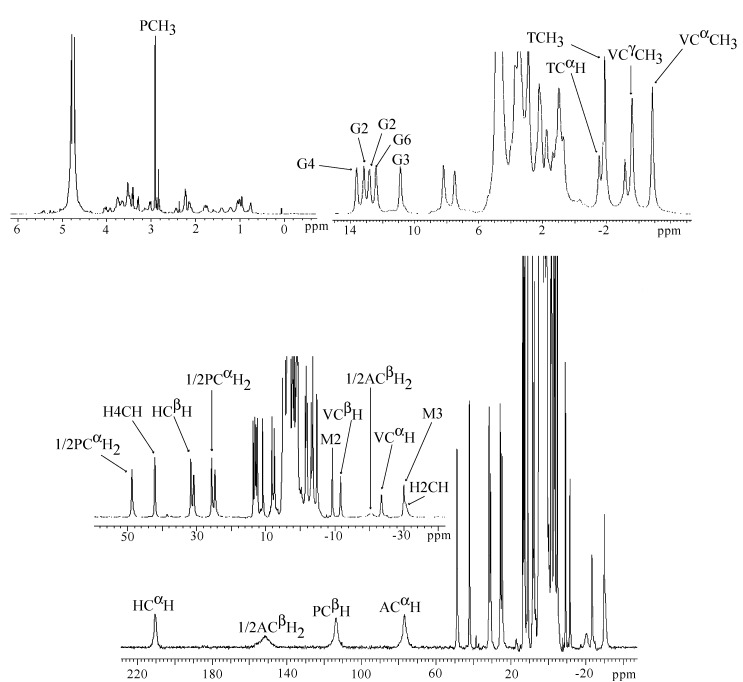
^1^H-NMR spectrum of Co(II)BLM [48].

**Figure 9:**
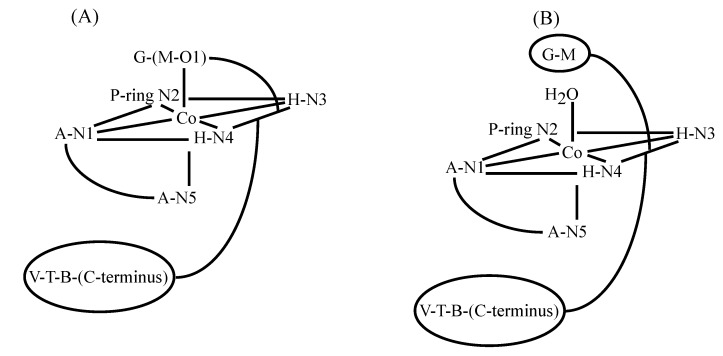
Coordination of Co(II) [48] to BLM-A_2_ and -B_2_ as proposed by Lehmann *et al*. The oxygen atom of the carbamoyl (**A**) or a solvent molecule (**B**) are bound as axial ligands.

Since the controversy involving the axial ligands (M carbamoyl group or A primary amine) had not been resolved through the investigations performed on HOO-Co(III)-bound BLMs to date, a study on the Co(III)BLM precursor, Co(II)BLM, was undertaken by Lehmann *et al.* [48]. The paramagnetic nature of this compound facilitated the identification of the ligands to the metal center. A combination of one- and two-dimensional NMR experiments was used to assign most of the ^1^H signals. The one-dimensional spectrum of Co(II)BLM with signal assignments is shown in Figure 8. The large chemical shifts exhibited by protons in the A, P, H, G, and M moieties hinted their closeness to the Co(II) center in this compound. The T_1_ relaxation times of these protons were used in MD calculations to determine the solution structure of Co(II)BLM. The MD results indicated that the structures that best fitted the NMR data collected for Co(II)BLM were six-coordinated, with the primary amine in A, and either the carbamoyl oxygen in M or a solvent molecule occupying the axial sites (Figure 9).

A natural-abundance ^15^N-NMR study of HOO-Co(III)BLM and its deglycosylated congener was carried out by Xia and co-workers, with the aim to test the possibility of axial coordination by the carbamoyl nitrogen in HOO-Co(III)BLM [49]. Using 2D ^1^H{^15^N} HSQC NMR experiments, it was possible to assign all of the ^15^N NMR signals of nitrogens with bound hydrogen in these compounds.

The ^15^N-NMR signals assigned to the M-NH_2_ nitrogen in *apo-*BLM was found not to be perturbed upon Co(III) complexation. Additionally, the positions of the signals generated by the A-NH and A-NH_2_ nitrogens in the HOO-Co(III) complexes were found to be highly shifted in comparison with the parent metal-free molecules. These two findings were used by the authors to state the identity of the endogenous axial ligand in HOO-Co(III)BLM as the A primary amine.

### 3.3. CuBLM Complexes

Interest in the structures of CuBLMs initially originated from the fact that BLMs are isolated as Cu(II) complexes. NMR studies of Cu(I)BLM and CO-Cu(I)BLM performed by Oppenheimer *et al*. [50] allowed the assignment of 13 NH signals. Among these signals, that of the amide nitrogen in H was identified. This finding indicated that the binding of Cu(I) did not require the coordination of N3. UV/visible spectroscopy was also used in this study to determine that the affinity of Cu(I) for BLM was at least 4-fold greater than that for Fe(II). This fact was used to suggest that Cu instead of Fe could be the biologically relevant metal center *in vivo*. More comprehensive NMR studies of Cu(I)- and CO-Cu(I)-bound BLM made possible the assignment of most of the ^13^C and ^1^H signals for this complex [51]. Changes in chemical shifts of resonances coming from protons in A, P and the imidazole in H upon binding of Cu(I), suggested the participation of these moieties in metal ligation. The presence of the signal elicited by the proton in HN3 (Figure 1) reinforced the hypothesis that N3 was not a ligand to the metal center in Cu(I)BLM, and suggested that this MBLM had a geometry different from that exhibited by Fe- and Zn-bound BLMs.

As a result of their studies on CuBLM some scientists support the hypothesis that this BLM adduct is a prodrug, delivering BLM to the cell nucleus where it becomes a spectator, and Fe(II) is responsible for the oxidative damage of DNA [52,53]. On the other hand, other research groups have delineated the conditions to demonstrate the activity of Cu(I)BLM when reductively activated [54]. It is important to determine if Cu(I)BLM is a prodrug, organizing the BLM residues to facilitate Fe(II) binding, or if this MBLM is oxidation-reductions active. 

**Figure 10:**
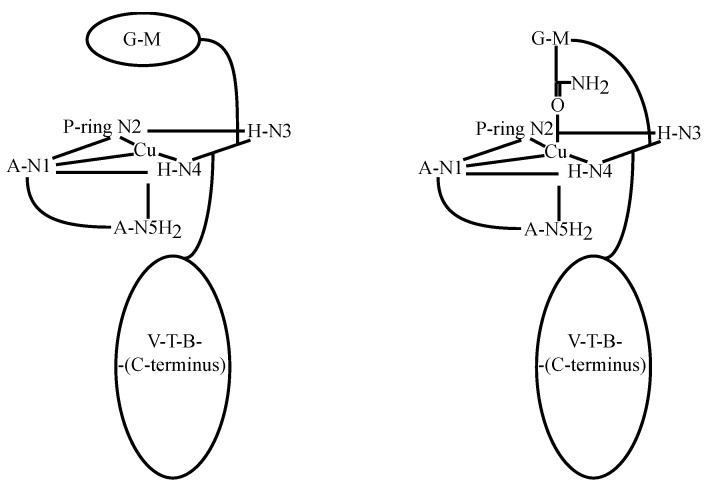
Cu(I)-coordination models proposed for Cu(I)BLM by Lehmann [55].

The NMR studies referred to above served as the starting point for the determination of the solution structure of Cu(I)BLM [55]. The assignment of all of the ^1^H signals was achieved in this study. Comparison of NMR one- and two-dimensional spectra collected for *apo-* and Cu(I)BLM indicated that the primary and secondary amines in A, N2 in the P moiety, and N4 in the imidazole ring were coordinated to Cu(I). As reported previously by others [50,51], a signal for the proton in HN3 was detected in this study, ruling out the coordination of N3 to the metal center. Analysis of the NOESY data collected for Cu(I)BLM evinced the lack of connectivities between protons in the coordination cage of the Cu(I) center (A, P, and H residues), and the V-T-B fragment. Additionally, the signals derived from the P-ring NH_2_ protons indicated that they were equivalent protons, and the unusual shifts of protons in the V unit, reported previously for other MBLMs were not observed for Cu(I)BLM. Collectively these three facts indicated that the DNA binding domain in Cu(I)BLM is not folded back underneath the equatorial plane of the metal as reported for HOO-Co(III)BLM (*vide supra*), and it was concluded that the Cu(I)BLM should exhibit an extended structure regarding the V-T-B-(C-terminus) fragment. Initial coordination models for Cu(I)BLM were built to be tested against the NMR data generated for this MBLM [55], considering different screw senses. Among all models considered, the ones shown in Figure 10 were the ones that best fitted the NMR data. Although not included in structural calculations, the V-T-B-(C-terminus) fragment in Cu(I)BLM was determined to have an extended conformation. This finding was used to support the hypothesis that Cu(I) center in Cu(I)BLM is not only helpful in delivering BLM to the cell nucleus, but it can also pre-organize the BLM residues before Fe(II) binding.

### 3.4. FeBLM Complexes

Although isolated as a Cu(II) complex [56], FeBLM is the one that has been implicated as the possible active species in BLM-directed DNA strand scission [5]. The paramagnetic nature of FeBLM compounds, which can broaden many ^1^H-NMR signals beyond detection, elicited the use of other MBLMs to pursue the coordination chemistry of MBLMs. In spite of the complications involved in the use of NMR to study paramagnetic complexes, various research groups reported studies of FeBLM through NMR. Examination of these studies shows how NMR was conducive to the identification of the BLM residues attached to the Fe ion in the most relevant MBLM. Additionally, the non-paramagnetic behavior of the bithiazole (B) and C-terminus fragments observed from the NMR spectra of Fe(II)BLM allowed scientists to determine that these two units are located far from the metal center in this complex. One of the first reports in this area is that by Dabrowiak *et al.* [57], where Fe(II)- and Fe(III)-bound BLM and TLM were examined through ^13^C-NMR. Although none of the paramagnetically-shifted ^1^H-NMR signals were assigned, a comparison of the spectra of *apo-* and FeBLM led to the conclusion that the P-H-G-M region in both antibiotics are utilized for binding both Fe(II) and Fe(III) ions. Changes in the ^13^C nuclear relaxation times (T_1_) that take place upon Fe(II) complexation were also used to identify some of the BLM ligands to the Fe ion [58]. The results of this investigation indicated that the longitudinal relaxation rates (1/T_1_) of the carbon atoms in the groups: A CH_2_, P CH_2_ and CH, and the carbon connected to the A primary amine; were the ones exhibiting sizable changes on complexation. These findings led to the proposal of the ion-chelation of the A primary amine and the P ring. Surprisingly, the imidazole ring was rule out as a coordinating ligand in this account.

**Figure 11:**
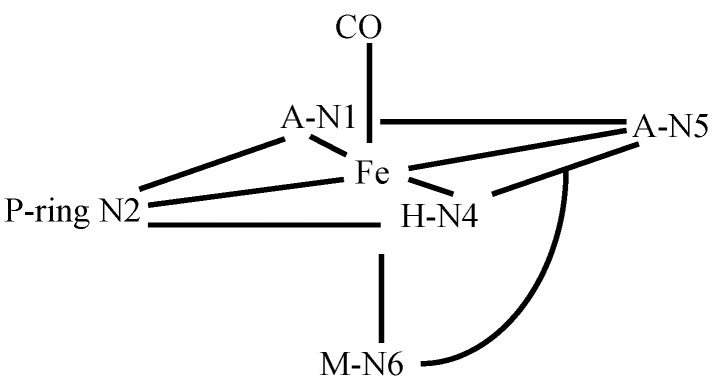
Arreangement of BLM lingndas in CO-Fe(II)BLM proposed by Oppenheimer *et al.* [59].

In order to alleviate the paramagnetic effects of the high spin Fe(II) metal center, CO-Fe(II)BLM complexes were studied [59]. CO-Fe(II)BLM are stable diamagnetic compounds, since binding of CO to the Fe(II) ion leads to a low spin CO-Fe(II)BLM complex. Fe(II)BLM NMR samples were examined in this work, and it was reported that ^1^H-NMR spectrum of Fe(II)BLM extended over a 70 ppm range, due to the paramagnetic influence of the Fe(II) ion. The rate of exchange between Fe(II)BLM and *apo*-BLM was reported to be too slow to allow the use of saturation transfer (ST) experiment, which could have allowed the assignment of the NMR signals. Both of these results would be challenged later by other NMR spectroscopists (*vide infra*). Addition of CO to Fe(II)BLM resulted in a well-resolved spectrum, similar to that of Zn(II)BLM. Nitrogens N1, N2, N4-N6 (Figure 1) were proposed to be the ligands to the metal center, together with CO. The proposed arrangement of the ligands around the Fe(II) center is shown in Figure 11.

ST experiments were performed by Pillai *et al*. on Fe(II)BLM for the first time in 1980 [60]. A Fe(II):BLM ratio of 1:2 and pH = 6.3 were used for the NMR sample preparation. The resulting NMR spectra exhibited signals elicited by Fe(II)BLM and its metal-free from, indicating that the two BLM forms are in fast exchange. The use ST allowed the assignment of some of the paramagnetically shifted signals in Fe(II)BLM, and led to the proposal of the binding of N2, N4, N5 and O1 (Figure 1). The P CONH_2_ and V OH or CO were also suggested as ligands. With this elegant work, the conditions to study Fe(II)BLM with more advanced NMR techniques were established.

The ^1^H- and ^13^C-NMR signals for the CO-Fe(II)BLM were fully assigned through two-dimensional NMR techniques by Akkerman and co-workers [61]. Inter-residue NOE data were used to determine the relative positions of the metal-binding sites, and these relative locations formed the basis for the determination of the first solution structure of CO-Fe(II)BLM. Nitrogen atoms N1 through N4 were postulated as ligands to Fe(II), together with the carbamoyl group in M. Interestingly, N2 was excluded as part of the first coordination sphere of Fe(II), although it was considered a ligand in Zn(II)BLM by the same research group [20]. The increase of the A CH-CH2 coupling constants in the CO-Fe(II)BLM complex when compared to those determined for Zn(II)BLM, which indicated that this BLM segment exhibited a flexibility similar to that of metal-free BLM, was used to eliminate N2 as a coordination point in CO-Fe(II)BLM.

As stated above, structural studies of non-biologically relevant MBLMs such as Z(II)- and Co(III)-bound adducts have been designed to circumvent the higher complexity involved in producing NMR samples and spectra of paramagnetic air-sensitive MBLMs. However, the *in vivo* catalytically relevant MBLM are air-sensitive and paramagnetic Fe-bound adducts. It is therefore required that these MBLMs be studied rather directly. The first step towards the elucidation of the solution structures of FeBLMs is to identify the atoms in the BLM molecule that are coordinated to the Fe(II) ion. NMR spectroscopy, with modified pulse sequences to fit paramagnetic molecules, was used to determine which atoms in the BLM molecule were influenced by the paramagnetic nature of the metal center [62]. The position of the NMR signals generated by some protons in the Fe(II)BLM complex determined from this investigation are presented in Table 1 below.

**Table 1:** Summary of the positions and relaxation times for the protons in Fe(II)BLM determined at 298 K.

Peak position (ppm)	Relaxation time T_1_ (ms)	Assignment
		β-hydroxyhistidine
206	1.2	C^α^H
121	<0.8	C2H
66.5	-	N3H
42.3	10.0	C4H
−17.3	3.1	C^β^H
		β-aminoalanine
204	2.8	C^β^H_2_
108	0.8	C^β^H_2_
127	1.5	C^α^H
		Pyrimidinilpropionamide
153	<0.8	C^β^H
44.9	7.9	C^α^H_2_
32.1	5.8	C^α^H_2_
2.1	-	CH_3_
14.0	-	CONH_2_
10.1	-	CONH_2_
		Methylvalerate
12.9	32.8	VALC^α^CH3
37.8	3.1	C^α^H
24.8	7.6	C^β^H
20.9	18.2	C^γ^H
8.1	23.5	C^γ^CH3
		Threonine
6.2	110.2	C^α^H
5.2	210.1	C^β^H
2.3	-	CH_3_
15.0	-	NH
		Gulose
−7.5	16.0	C1
−4.7	59.4	C2
−2.36	-	C3
3.6	-	C4
−5.4	15.3	C5
1.5	106.0	C6
2.3	115.0	C6
		Mannose
−1.38	78.8	C1
−2.10	90.9	C2 or C3
−2.40	106.5	C2 or C3
−2.48	111.8	C4
−12.5	22.1	C5
−2.23	141.0	C6
−2.68	133.3	C6

As can be seen from Table 1, most the NMR signals generated by the protons in H, A, and P display chemical shifts very different from those expected for the same protons in a diamagnetic molecule (0–10 ppm). These unusual chemical shifts are attributed to close proximity of these protons to the Fe(II) center. This fact is also supported by the short relaxation times exhibited by these protons, also shown in Table 1 (T_1_s in the order of seconds are expected for a diamagnetic molecule). This set of results supported the fact that the H, A, and P residues in BLM, which are part of the metal binding domain of the molecule, are ligated to the metal center through N1, N2, N3, N4, and N5 (Figure 1), as previously reported by other investigations. The chemical shifts of the disaccharide are also atypical for a diamagnetic molecule, 4–6 ppm expected for these protons in the absence of Fe(II). However, these chemical shifts and their corresponding T_1_s are similar to those exhibited by some protons in the threonine (T) and V residues, which have been shown not to coordinate to the metal center in MBLMs. At the conclusion of this research work, it was possible to establish that N1, N2, N3, N4, and N5 were coordinating ligands, and had some evidence hinting the metal coordination of the disaccharide segment. However, additional work was required to either support or discard disaccharide coordination. 

A solution structure, compatible with the NMR data referred to above, is required to better understand the way the BLM molecule wraps its residues around the Fe(II) center in this MBLM. Additionally, this solution structure can serve to test the possible coordination of the disaccharide fragment of BLM to the Fe(II) ion. MD calculations were performed on initial models built for Fe(II)BLM. These initial models were constructed based on the findings of Loeb and co-workers while investigating the coordination chemistry of Fe(II)BLM through magnetic circular dichroism [63]. The results of Loeb’s research indicated that the Fe(II) ion is six-coordinated in Fe(II)BLM through N1, N2, N3, N4, and N5; with a sixth coordination site proposed to be either O1 or a solvent molecule. Based on these results, five initial models for Fe(II)BLM were built considering all possible screw senses, and involving a water molecule or O1 as the sixth ligand [64]. Usually NOE and J-coupling constant information are used to test plausible initial models against NMR data generated for them. However, Fe(II)BLM is a highly paramagnetic molecule, yielding almost undetectable non-trivial NOEs; and exhibits very broad NMR signals, preventing the determination of J values. One way around this problem is to calculate proton-to-metal distances, determined from the T_1_ values measured for the protons in Fe(II)BLM, to use them in MD calculations instead of NOE and J-coupling data. This strategy was used in MD calculations performed on the five initial models referred to above, and yielded two structures that fitted the NMR constraints imposed and had the lowest energies. These solution conformation turned out to be isostructural with those of Co(II)BLM (Figure 9) [48]. These structures show that nitrogens N1, N2, N3, and N4 are equatorially coordinated to Fe(II); and N5 and either O1 or a water molecule can be coordinated axially. They also showed how the BLM residues are organized around the Fe(II) center in this MBLM. However, since the energetics of molecules bearing different ligands cannot be compared in a one-to-one fashion, it was not possible in this study to rule out any of the two possible sixth ligands.

**Scheme 1:**
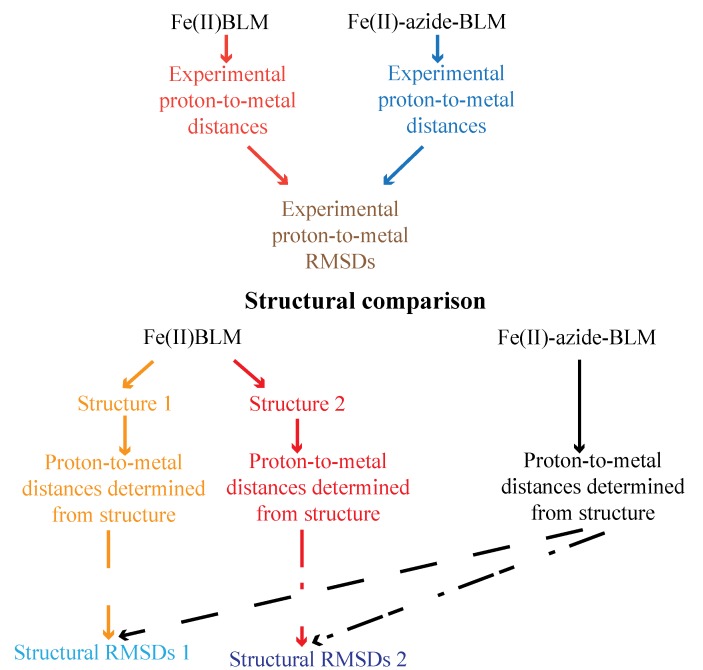
Flow chart of the structural comparisons performed between Fe(II)BLM and Fe(II)-azideBLM.

A reasonable proposal of coordinated ligands is required before the solution structures of MBLM-DNA complexes are determined. Therefore, it is necessary that the ambiguity regarding the sixth ligand be resolved. Loeb *et al*. determined that in the presence of azide the sixth ligand in Fe(II)BLM is released from coordination to allow azide binding [63]. With the aim of either supporting or not the coordination of a water molecule to the metal center in Fe(II)BLM, derived the solution structure of Fe(II)-azide-BLM was determined from NMR data [65,66]. The structural changes that take place on Fe(II)BLM upon azide binding were monitored by comparing the differences (root mean square deviations (RMSD)) between the experimentally determined proton-to-metal distances for Fe(II)BLM and Fe(II)-azide-BLM with those obtained from the calculated structures for Fe(II)BLM (structures 1 and 2) and Fe(II)-azide-BLM (Scheme 1). Comparison of the experimental proton-to metal RMSDs with the structural RMSDs 1 and 2 indicated that the experimental Fe(II)BLM → Fe(II)-azide-BLM transition was better represented by the Fe(II)BLM (Structure 2) → Fe(II)-azide-BLM conversion. These results allowed ruling out the coordination of a water molecule to the Fe(II) ion, and proposing the disaccharide fragment as the sixth ligand through O1.

**Figure 12:**
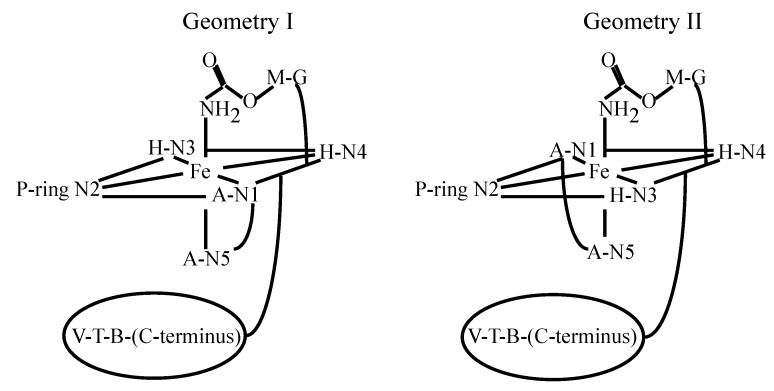
Coordination geometries tested for Fe(II)PEP [67].

NMR-derived solution structures of Fe(II)-bound PEP have also been reported [67]. Previous investigations of the solution structures of MBLM led to the proposal of two possible coordination geometries for these complexes (Figure 12). These two geometries were tested against NMR data through MD calculations in this study. The outcome of this investigation indicated that both of them were equally likely in solution for Fe(II)PEP in the absence of DNA. On the other hand, the crystal structure of HOO-Co(III)BLM bound to DNA fragments indicate that it is geometry II (Figure 12) the one favored by HOO-Co(III)BLM in the presence of DNA [11]. An examination of the hydrogen-bond network required to stabilize the (HOO-Co(III)BLM)-DNA structure was used to explain this fact. 

In summary, NMR studies performed directly on Fe(II)BLMs have (1) allowed the identification of the ligands to the metal center in Fe(II)BLM through the paramagnetic character of the BLM moieties containing them; (2) generated solution structures for Fe(II)BLM compatible with the NMR data generated for this complex, exhibiting the organization of the BLM residues around the metal center; and (3) identified both axial ligands in the coordination sphere of the Fe(II) ion.

## 4. Metallo-BLM Interactions with DNA

Indubitably NMR has been the key spectroscopic technique used in studies of MBLM-DNA interactions. Nonetheless, the mode of binding of MBLM to DNA remains unclear. Previous structural studies involving MBLMs and MBLM-DNA complexes have allowed the identification of the B-(C-terminus) segment in this molecule as the one that more closely interacts with DNA (Figure 1). However, in most cases each of these studies has been undertaken using a different DNA fragment, selected with specific goals in mind, without openly considering that the binding of MBLMs to DNA depends on the DNA base sequence. Additionally, the research aimed to determine MBLMs interactions with DNA has also randomly employed BLMs with different tails (BLM-A_2_, -B_2_, -A_5_, PEP, *etc*.), but has only focused on the B segment, disregarding the influence that the BLM C-terminus might have in the mode of binding of MBLMs to DNA and in BLM pulmonary toxicity. Since most of the structural studies on MBLMs and MBLM-DNA complexes use NMR, and the biologically relevant MBLM, Fe(II)BLM, is a paramagnetic complex; these studies have used non-biologically relevant, Co(III)- and Zn(II)-bound, BLMs to avoid paramagnetic samples that affect NMR spectra to a great extent. Pseudo-activated MBLMs and non-active MBLMs such as HOO-Co(III)- and Zn(II)BLMs, respectively, have been investigated and the results of these investigations have been compared without regard for the capabilities of these MBLMs to cleave DNA. Solution structures of MBLM-DNA complexes are available in the literature [43,68,69,70,71,72,73,74,75,76,77]. However, these structures have resulted from individual studies of these complexes each one using a different non-biologically relevant MBLM (HOO-Co(III)BLM-A_2_, Zn(II)BLM-A_5_, HOO-Co(III)PEP, HOO-Co(III) BLM-B_2_, *etc*.); interacting with different DNA fragments in each study (d(CCAGGCCTGG), d(CGCTAGCG), d(CGTACG), d(ATTAGTTATAACTAAT), *etc*.). The results of these studies have led to the proposals of three different modes of binding of MBLMs to DNA: partial or total intercalation of the B unit between DNA bases, or binding to the minor groove.

Evidence in support of the three models for the binding of BLMs to DNA has accumulated over time. Povirk and co-workers [78] used fluorometry, equilibrium dialysis, electric dichroism, temperature-jump, and stopped-flow spectrometry to study the binding of Cu(II) and Fe(III) complexes of the clinical mixture of BLMs, blenoxane (70% BLM-A_2_, 30% BLM-B_2_), to calf thymus DNA and Co1E1 DNA. The results of this investigation indicated that a lengthening of DNA between 3.2 and 4.6 Å occurred upon binding, which are also exhibited by known DNA intercalators. Additionally, Urata *et al*. studied the binding of Zn(II)BLM-B_2_ to L- and D-d(CGCGCG)_2_ through NMR [79]. A large upfield shift of the bithiazole non-exchangeable protons in BLM (0.2–0.6 ppm) was observed upon binding to the DNA fragments, which is within the range of typical intercalators (0.4–1.0 ppm). NMR studies of HOO-Co(III)BLM-A_2_ bound to the DNA oligomer d(CCAAAGXACTGGG).d(CCCAGTACTTTGG), where X represents a 3′-phosphoglycolate lesion next to a 5′-phosphate, [69] also support the model of total intercalation through the NMR-derived solution structures for this MBLM-DNA complex. The crystal structures of HOO-Co(III)BLM-B_2_ bound to d(ATTAGTTTAACTAAT) and d(ATTAGTTATAACTAAT), binding sites underlined, hinted two distinct modes of full intercalation of the B fragment to the DNA segments, stabilized by different sets of hydrogen bonds [11].

Results conflicting with the total intercalation model came from studies of the angle of unfolding of supercoiled Co1E1 DNA in the presence of metal-free blenoxane [78]. A value of 12° was determined in this case, whereas for the majority of intercalators it constitutes 17–26°. Changes in the ^1^H NMR spectrum of Zn(II)BLM-A_2_ upon its binding to poly(dA-dT) were incompatible with the complete intercalation model [80]. Although the most pronounced changes are observed for the signals of the B segment (upfield shifts between 0.1–0.2 ppm), for typical intercalators this varies from 0.4 to 1.0 ppm. Similar results were observed when ^1^H-NMR was used to study the binding of BLM-A_2_ to poly (dA–dT) [81,82,83], demethyl-BLM-A_2_ to poly(dA-dT) [83], and BLM-A_2_ to sonicated calf thymus DNA [84]. The binding of Ni(II)- and VO(III)- bound BLM-B_2_ to the duplex d(CCCCAGCTGGGG) produced upfield chemical shifts changes between 0.15–0.2 ppm. No changes in the chemical shifts of the signals of the imino protons and phosphorus nuclei of the oligonucleotide in the vicinity of the binding site were observed [85]. However, it is known that intercalating molecules produce significant changes in the chemical shifts of the signals of these nuclei. NMR spectroscopy was also used in the investigation of the binding of HOO-Co(III)BLM-A_2_ to DNA duplexes d(CCAGGCCTGG)_2_ [73,75] and d(CCAGTACTGG) [72], H_2_O-Co(III)BLM-A_2_ bound to d(CCAGGCCTGG) [70]; HOO-Co(III)-deglyco-BLM-A_2_ bound to d(CCAGGCCTGG) [76]; and HOO-Co(III)-deglyco-PEP bound to the oligonucleotide CGTACG [70], The results of these investigations indicated that the terminal thiazole ring is well stacked within the duplex DNA, while the penultimate thiazole ring is only partially stacked. In order to rationalize the experimental data in favor of total intercalation and against it the partial intercalation model was suggested. In this model one of the B rings is inserted between the DNA bases, thereby producing local bending of the helix. Partial intercalation also provides an explanation for relatively small changes in the chemical shifts of the B protons. 

Changes in site-specificity and efficiency of DNA cleavage by BLM were observed in studies involving ligands tightly bound to the minor groove of DNA, supporting the hypothesis of BLM binding to the minor groove of DNA through its B-(C-terminus) segment. These studies included binding and cleavage of a 327-base-pair segment pBR322 DNA by FeBLM in the presence of ethidium bromide, distamycin A and actinomycin D [86]; and various fragments of pbcNI chromosomal DNA by FePEP in the presence distamycin A and other minor groove binders such as Hoechst 33258, DAPI, and berenil [87]. On the other hand, covalent attachment of aflatoxin B to the N(7) atom of guanine or of mitomycin C to the O(6) atom of guanine in the major groove of 220- and 327-base pair pBR322 DNA fragments had practically no influence on the specificity or efficiency of DNA cleavage by FeBLM [88] Two-dimensional NMR data also testify to the possibility of BLM binding in the minor groove of the double helix. NMR studies of the binding of Zn(II)BLM-A_5_ to the 8-meric duplex d(CGCTAGCG)_2_ indicated that the sequential NOE connectivities of base and sugar protons of the DNA fragment were not disrupted by the binding of this MBLM. NMR-based molecular dynamics calculations of the structure of Zn(II)BLM-A_5_ bound to the DNA fragment indicated that the BLM-A_5_ molecule is localized in the minor groove of DNA, overlapping 4–5 base pairs [71].

Collectively, these three bodies of information, together with respective additional results derived from studies of DNA binding by model complexes of the B-(C-terminus) segments of BLM in each case; support either the total or partial intercalation, or binding to the minor groove of DNA. Although the extensive work cited above provides convincing evidence for each mode of binding of MBLMs to DNA, factors such as: (1) the metal centers, their activated and non-activated states, and different coordination chemistries; (2) BLMs with different C-termini; and (3) different DNA specimens are used in these studies indiscriminately, which makes it difficult to compare the results in a conclusive fashion. Additionally, all the structural work performed involves only non-biologically relevant MBLMs. The body of work reviewed in this section makes it clear that there is a critical need to establish the extent to which the BLM tail is a factor guiding the binding of MBLMs to DNA. It is also necessary to concentrate on biologically relevant MBLMs. Previous research work supports the need to address the mode of DNA binding by MBLMs containing the catalytically relevant Fe(II) site [89].

## 5. Conclusions

The crystal structure of DNA-bound Co(III)BLM-B_2_ reported by Goodwin *et al*. [11] has confirmed the equatorial ligands previously identified through the NMR studies discussed above. Additionally, the axial coordination of the A-NH_2_ nitrogen was also supported by this structure. Although it is now well known that the M-carbamoyl group is not ligated to Co(III) in the (HOO-Co(III)BLM)-DNA complex, its coordination to Co(II) or Fe(II) in their complexes with BLMs should not be ruled out. The crystal structure of DNA-bound Co(III)BLM-B_2_ [11] indicates that the gulopyranose moiety serves as a space-filling unit allowing the metal binding domain to adopt an optimized and stabilized orientation relative to the target DNA. If the G-M segment of BLM is to partially stack with the metal binding domain upon DNA binding, it makes sense that it remains in the vicinity of the metal after its possible release from coordination. The results of studies performed on Fe(II)BLM [48,62,64,65] suggest that it is possible that the role of the disaccharide moiety in BLM changes as the process that involves MBLM formation, oxygen activation, and DNA binding and cleavage follows its course. A possible scheme of events with different roles for the gulopyranose fragment could be: (1) initial weak binding of the carbamoyl to the deoxygenated Fe(II) center assists BLM in its competition for an effective sequestration of intracellular iron prior to a ligand structural reorganization and oxygenation; (2) in the presence of oxygen, the carbamoyl group is released from coordination to allow O_2_ binding and activation; (3) after O_2_ binding, the role of the disaccharide unit is that of stabilizing the MBLM-DNA complex through space-filling and hydrogen bonding.

Regarding the interactions of MBLMs with DNA, a detailed structural characterization of MBLM-DNA complexes through NMR involving only biologically-relevant MBLMs, separating the BLM tail and the DNA base sequence as independent important factors in DNA binding by BLMs, and taking into account the activation state of the metal and the degree of pulmonary toxicity of each BLM considered is required. In the medical field, big research efforts are devoted each year toward eliminating or alleviating pulmonary fibrosis caused by BLM in cancer patients through combination chemotherapy. Although some of these efforts have produced successful outcomes, the results are no general enough to be applied to a wide cancer spectrum [90]. The connection between the BLM tail and pulmonary fibrosis established by Raisfeld and others through experimental animal model studies [91,92,93] has supported the development of new BLMs with less toxic tails. Since the introduction of the clinically used mixture of BLMs, blenoxane, to clinical medicine in 1972, attempts have been made at modifying the basic BLM structure at the C-terminus to improve its therapeutic index. However, other than their demonstrated role in binding to DNA, the pharmacological and toxicologic importance of particular C-termini on BLM remains unclear. It can be expected that detailed structural characterization at the molecular level of BLM binding to DNA and the structure/toxicity connection can unveil even more intimate details of the MBLM-DNA interactions. NMR should be the tool of choice to achieve this goal for various reasons: (1) Biologically relevant FeBLM compounds can be characterized with it; (2) Unlike other spectroscopic techniques, NMR allows the monitoring of the whole MBLM-DNA system; (3) NMR data can be transformed into MBLM-DNA solution structures.

## Supplementary Materials

Supplementary File 1
