# Supplementary material for: Contributions of NMR to the Understanding of the Coordination Chemistry and DNA Interactions of Metallo-Bleomycins
Source: Molecules. 2013 Aug 2;18(8):9253–77. doi: 10.3390/molecules18089253 (PMC6270211; doi:10.3390/molecules18089253)
Supplement: Supplementary File 1 [file molecules-18-09253-s001.docx]

Correction of Reference list

References

1. Bennett, J.M.; Reich, S.D. Bleomycin. *Ann. Inter. Med.* 1979, *90*, 945–948.
2. Carlson, R.W.; Sikic, B.I.; Turbow, M.M.; Ballon, S.C. Combination cisplatin, vinblastine, and bleomycin chemotherapy (pvb) for malignant germ-cell tumors of the ovary. *J. Clin. Oncol.* 1983, *1*, 645–651.
3. Sikic, B.I.; Rozencweig, M.; Carter S.K. *Bleomycin Chemotherapy*; Academic Press: Orlando, FL, USA, 1985.
4. Einhorn, L.H.; Donohue, J. Cis-diamminedichloroplatinum, vinblastine, and bleomycin combination chemotherapy in disseminated testicular cancer. *Ann. Inter. Med.* **1977**, *87*, 293–298.
5. Sausville, E.A.; Peisach, J.; Horwitz, S.B. Role for ferrous ion and oxygen in degradation of DNA by bleomycin. *Biochem. Biophys. Res. Commun.* 1976, *73*, 814–822.
6. Hecht, S.M. RNA degradation by bleomycin, a naturally-occurring bioconjugate. *Bioconjugate Chem.* 1994, *5*, 513–526.
7. Kane, S.A.; Hecht, S.M. Polynucleotide recognition and degradation by bleomycin. *Prog. Nucleic Acid Res. Mol. Biol.* 1994, *49*, 313–352.
8. Hecht, S.M. Bleomycin: New perspectives on the mechanism of action. *J. Nat. Prod.* **2000**, *63*, 158–168.
9. Iitaka, Y.; Nakamura, H.; Nakatani, T.; Muraoka, Y.; Fujii, A.; Takita, T.; Umezawa, H. Chemistry of bleomycin. XX. X-ray structure determination of P-3A Cu(II)-complex, a biosynthetic intermediate of bleomycin. *J. Antibiot.* **1978**, *31*, 1070–1072.
10. Sugiyama, M.; Kumagai, T.; Hayashida, M.; Maruyama, M.; Matoba, Y. The 1.6-Å crystal structure of the copper(II)-bound bleomycin complexed with the bleomycin-binding protein from bleomycin-producing Streptomyces verticillus. *J. Biol. Chem.* **2002**, *277*, 2311–2320.
11. Goodwin, K.D.; Lewis, M.A.; Long, E.C.; Georgiadis, M.M. Crystal structure of DNA-bound Co(III)-bleomycin B-2: Insights on intercalation and minor groove binding. *Proc. Natl. Acad. Sci. USA* **2008**, *105*, 5052–5056.
12. Takita, T.; Muraoka, Y.; Yoshioka, T.; Fujii, A.; Maeda, K.; Umezawa, H. Chemistry of bleomycin .9. Structures of bleomycin and phleomycin. *J. Antibiot*. **1972**, *25*, 755–758
13. Naganawa, H.; Muraoka, Y.; Takita, T.; Umezawa, H. Chemistry of bleomycin. 18. C-13
    NMR-studies. *J. Antibiot*. **1977**, *30*, 388–396.
14. Naganawa, H.; Takita, T.; Umezawa, H.; Hull, W.E. Chemistry of bleomycin. 23. Natural abundance N-15-NMR spectroscopic evidence for the structure of bleomycin. *J. Antibiot.* **1979**, *32*, 239–241.
15. Umezawa, H. Structure and action of bleomycin. *Prog. Biochem. Pharmacol.* 1976, *11*, 18–27.
16. Chen, D.; Hawkins, B.L.; Glickson, J.D. Proton nuclear magnetic-resonance study of bleomycin in aqueous-solution-assignment of resonances. *Biochemistry* **1977**, *16*, 2731–2738.
17. Haasnoot, C.A.G.; Pandit, U.K.; Kruk, C.; Hilbers, C.W. Complete assignment of the 500 MHz H-1-NMR spectra of bleomycin A2 in H_2_O and D_2_O solution by means of two-dimensional NMR-spectroscopy. *J. Biomol. Struct. Dyn.* **1984**, *2*, 449–467.
18. Tanaka, W.; Takita, T. Pepleomycin-2nd generation bleomycin chemically derived from bleomycin A_2_. *Heterocycles* **1979**, *13*, 469–476.
19. Oka, S. A review of clinical-studies of peplomycin. *Recent Res. Cancer* **1980**, *74*, 163–171.
20. Lehmann, T.; Li, Y.; Armstrong, G.S. NMR study of peplomycin in aqueous solution. Assignment of resonances by means of two-dimensional spectroscopy. *J. Antibiot*. **2011**, *64*,
    309–316.
21. Dabrowiak, J.C.; Greenaway, F.T.; Longo, W.E.; Vanhusen, M.; Crooke, S.T. Spectroscopic investigation of metal-binding site of bleomycin A-2-Cu(II) and Zn(II) derivatives.
    *Biochim. Biophys. Acta* **1978**, *517*, 517–526.
22. Cass, A.E.G.; Galdes, A.; Hill, H.A.O.; Mcclelland, C.E. Binding of Zinc(II) to
    bleomycin-investigation using H-1 NMR-spectroscopy. *FEBS Lett*. **1978**, *89*, 187–190.
23. Dabrowiak, J.C.; Greenaway, F.T.; Grulich, R. Transition-metal binding-site of bleomycin
    A2-C-13 nuclear magnetic-resonance study of Zinc(II) and Copper(II) derivatives. *Biochemistry* **1978**, *17*, 4090–4096.
24. Lenkinski, R.E.; Dallas, J.L. An NMR investigation of the kinetics of dissociation of the Zinc(II) complex of bleomycin antibiotics. *J. Am. Chem. Soc.* **1979**, *101*, 5902–5906.
25. Oppenheimer, N.J.; Rodriguez, L.O.; Hecht, S.M. Proton nuclear magnetic-resonance study of the structure of bleomycin and the Zinc-bleomycin complex. *Biochemistry* **1979**, *18*, 3439–3445.
26. Oppenheimer, N.J.; Rodriguez, L.O.; Hecht, S.M. Metal-binding to modified bleomycins-zinc and ferrous complexes with an acetylated bleomycin. *Biochemistry* **1980**, *19*, 4096–4103.
27. Akkerman, M.A.J.; Haasnoot, C.A.G.; Hilbers, C.W. Studies of the solution structure of the bleomycin-A2 zinc complex by means of two-dimensional NMR-spectroscopy and distance geometry calculations. *Eur. J. Biochem.* **1988**, *173*, 211–225.
28. Akkerman, M.A.J.; Haasnoot, C.A.G.; Hilbers, C.W. Complete assignment of the C-13
    NMR-spectra of bleomycin-A2 and its zinc complex by means of two-dimensional
    NMR-spectroscopy. *Magn. Reson. Chem.* **1988**, *26*, 793–802.
29. Williamson, D.; Mclennan, I.J.; Bax, A.; Gamcsik, M.P.; Glickson, J.D. Two-dimensional
    NMR-study of bleomycin and its zinc(II) complex-reassignment of C-13 resonances. *J. Biomol. Struct. Dyn.* **1990**, *8*, 375–398.
30. Calafat, A.M.; Won, H.; Marzilli, L.G. A new arrangement for the anticancer antibiotics tallysomycin and bleomycin when bound to zinc: An assessment of metal and ligand chirality by NMR and molecular dynamics. *J. Am. Chem. Soc.* **1997**, *119*, 3656–3664.
31. Poulouse, K.P.; Watkins, A.E.; Reba, R.C.; Eckelman, W.C.; Goodyear, M. Cobalt-labeled bleomycin-new radiopharmaceutical for tumor localization-comparative clinical evaluation with gallium citrate. *J. Nucl. Med.* **1975**, *16*, 839–841.
32. Lin, M.S.; Goodwin, D.A.; Kruse, S.L. Bleomycin as a TC-99M carrier in tumor visualization.
    *J. Nucl. Med.* **1974**, *15*, 338–342.
33. De Reimer, L.H.; Meares, C.F.; Goodwin, D.A.; Diamanti, C.I. BLEDTA–tumor-localization by a bleomycin analog containing a metal-chelating group. *J. Med. Chem.* **1979**, *22*, 1019–1023.
34. Sugiura, Y. Bleomycin-iron complexes-electron-spin resonance study, ligand effect, and implication for action mechanism. *J. Am. Chem. Soc.* **1980**, *102*, 5208–5215.
35. Bereman, R.D.; Winkler, M.E. Coordination chemistry of the anti-tumor compound bleomycin-a spectral investigation of the cobalt complex. *J. Inorg. Nucl. Chem.* **1980**, *42*, 1797–1799.
36. Kakinuma, J.; Orii, H. Preparation, isolation and identification of bleomycin Co-57 chelate with special reference to its chelate structure. *J. Labelled Compd. Rad.* **1979**, *16*, 200.
37. Vos, C.M.; Westera, G.; Vanzanten, B. Different forms of the cobalt-bleomycin A2 complex.
    *J. Inorg. Biochem.* **1980**, *12*, 45–55.
38. Vos, C.M.; Westera, G.; Schipper, D. A C-13 NMR and electron-spin-resonance study on the structure of the different forms of the cobalt-bleomycin A2 complex. *J. Inorg. Biochem.* **1980**, *13*, 165–177.
39. Tsukayama, M.; Randall, C.R.; Santillo, F.S.; Dabrowiak, J.C. Transition-metal binding-site of bleomycin-Cobalt(III) bleomycin. *J. Am. Chem. Soc.* **1981**, *103*, 458–461.
40. Dabrowiak, J.C.; Tsukayama, M. Cobalt(III) complex of pseudotetrapeptide-A of bleomycin.
    *J. Am. Chem. Soc.* **1981**, *103*, 7543–7550.
41. Xu, R.; Antholine, W.E.; Petering, D.H. Reaction of Co(II)bleomycin with dioxygen. *J. Biol. Chem.* **1992**, *267*, 944–949.
42. Xu, R.X.; Nettesheim, D.; Otvos, J.D.; Petering, D.H. NMR determination of the structures of peroxycobalt(III) bleomycin and cobalt(III) bleomycin, products of the aerobic oxidation of cobalt(II) bleomycin by dioxygen. *Biochemistry* **1994**, *33*, 907–916.
43. Wu, W.; Vanderwall, D.E.; Lui, S.M.; Tang, X.J.; Turner, C.J.; Kozarich, J.W.; Stubbe, J. Studies of Co center dot bleomycin A2 green: Its detailed structural characterization by NMR and molecular modeling and its sequence-specific interaction with DNA oligonucleotides. *J. Am. Chem. Soc.* **1996**, *118*, 1268–1280.
44. Carter, S.K.; Ultmann, J. Peplomycin. *Cancer Treat. Rev.* **1984**, *11*, 303–305.
45. Takahashi, K.; Ekimoto, H.; Aoyagi, S.; Koyu, A.; Kuramochi, H.; Yoshioka, O.; Matsuda, A.; Fujii, A.; Umezawa, H. Biological studies on the degradation products of 3-[(s)-1′-phenylethylamino]propylaminobleomycin-novel analog (pepleomycin). *J. Antibiot.* **1979**, *32*, 36–42.
46. Matsuda, A.; Yoshioka, O.; Ebihara, K.; Yashamita, T.; Umezawa, H. *Bleomycin: Current Status and New Developments*; Academic Press: New York, NY, USA, 1978; pp. 299–331.
47. Caceres-Cortes, J.; Sugiyama, H.; Ikudome, K.; Saito, I.; Wang, A.H.J. Structures of cobalt(III)-pepleomycin and cobalt(III)-deglycopepleomycin (green forms) determined by NMR studies.
    *Eur. J. Biochem.* **1997**, *244*, 818–828.
48. Lehmann, T.E.; Serrano, M.L.; Que, L. Coordination chemistry of Co(II)-bleomycin: Its investigation through NMR and molecular dynamics. *Biochemistry* **2000**, *39*, 3886–3898.
49. Xia, C.W.; Forsterling, F.H.; Petering, D.H. Identification of the internal axial ligand of
    HO2-cobalt(III)-bleomycin: H-1{N-15} HSQC NMR investigation of bleomycin, deglycobleomycin, and their hydroperoxide-cobalt(III) complexes. *Biochemistry* **2003**, *42*, 6559–6564.
50. Oppenheimer, N.J.; Chang, C.; Rodriguez, L.O.; Hecht, S.M. Copper(I).bleomycin-a structurally unique oxidation-reduction active complex. *J. Biol. Chem.* **1981**, *256*, 1514–1517.
51. Ehrenfeld, G.M.; Rodriguez, L.O.; Hecht, S.M.; Chang, C.; Basus, V.J.; Oppenheimer, N.J. Copper(I)-bleomycin-structurally unique complex that mediates oxidative DNA strand scission. *Biochemistry* **1985**, *24*, 81–92.
52. Takahashi, K.; Yoshioka, O.; Matsuda, A.; Umezawa, H. Intracellular reduction of cupric ion of bleomycin copper complex and transfer of cuprous ion to a cellular protein. *J. Antibiot.* **1977**, *30*, 861–869.
53. Suzuki, T.; Kuwahara, J.; Sugiura, Y. Copper-bleomycin has no significant DNA cleavage activity. *Biochemistry* **1985**, *24*, 4719–4721.
54. Ehrenfeld, G.M.; Shipley, J.B.; Heimbrook, D.C.; Sugiyama, H.; Long, E.C.; Vanboom, J.H.; Vandermarel, G.A.; Oppenheimer, N.J.; Hecht, S.M. Copper-dependent cleavage of DNA by bleomycin. *Biochemistry* **1987**, *26*, 931–942.
55. Lehmann, T.E. Structural study of copper(I)-bleomycin. *J. Biol. Inorg. Chem.* **2004**, *9*, 323–334.
56. Umezawa, H.; Maeda, K.; Takeuchi, T.; Okami, Y. New antibiotics bleomycin A and B.
    *J. Antibiot.* **1966**, *19*, 200–209.
57. Dabrowiak J.C.; Greenaway, F.T.; Santillo, F.S.; Crooke, S.T. The iron complexes of bleomycin and tallysomycin. *Biochem. Biophys. Res. Commun.* **1979**, *91*, 721–729.
58. Gupta, R.K.; Ferretti, J.A.; Caspary, W.J. Location of iron in the Fe^2+^-bleomycin complex as observed by C-13 NMR-spectroscopy. *Biochem. Biophys. Res. Commun.* **1979**, *89*, 534–541.
59. Oppenheimer, N.J.; Rodriguez, L.O.; Hecht, S.M. Structural studies of active complex of bleomycin-assignment of ligands to the ferrous ion in a ferrous-bleomycin carbon monoxide complex. *Proc. Natl. Acad. Sci. USA* **1979**, *76*, 5616–5620.
60. Pillai R.P.; Lenkinski, R.E.; Sakai, T.T.; Geckle, J.M.; Krishna, N.R.; Glickson, J.D. Proton NMR-study of iron(II)-bleomycin: Assignment of resonances by saturation transfer experiments. *Biochem. Biophys. Res. Commun.* **1980**, *96*, 341–349.
61. Akkerman, M.A.J.; Neijman, E.W.J.F.; Wijmenga, S.S.; Hilbers, C.W.; Bermel, W. Studies of the solution structure of the bleomycin-A2 iron(II) carbon-monoxide complex by means of
    2-dimensional NMR-spectroscopy and distance geometry calculations. *J. Am. Chem. Soc.* **1990**, *112*, 7462–7474.
62. Lehmann, T.E.; Ming, L.J.; Rosen, M.E.; Que, L. NMR studies of the paramagnetic complex Fe(II)-bleomycin. *Biochemistry* **1997**, *36*, 2807–2816.
63. Loeb, K.E.; Zaleski, J.M.; Hess, C.D.; Hecht, S.M.; Solomon, E.I. Spectroscopic investigation of the metal ligation and reactivity of the ferrous active sites of bleomycin and bleomycin derivatives. *J. Am. Chem. Soc.* **1998**, *120*, 1249–1259.
64. Lehmann, T.E. Molecular modeling of the three-dimensional structure of Fe(II)-bleomycin: Are the Co(II) and Fe(II) adducts isostructural? *J. Biol. Inorg. Chem.* **2002**, *7*, 305–312.
65. Lehmann, T.E.; Li, Y. Possible structural role of the disaccharide unit in Fe-bleomycin before and after oxygen activation. *J. Antibiot.* **2012**, *65*, 25–33.
66. Lehmann, T.E.; Li, Y. Solution structure of Fe(II)-azide-bleomycin derived from NMR data: Transition from Fe(II)-bleomycin to Fe(II)-azide-bleomycin as derived from NMR data and structural calculations. *J. Biol. Inorg. Chem.* **2012**, *17*, 761–771.
67. Lehmann, T.; Li, Y. Coordination chemistry and solution structure of Fe(II)-peplomycon. Two possible coordination geometries. *J. Inorg. Biochem.* **2012**, *111*, 50–58.
68. Caceres-Cortes, J.; Sugiyama, H.; Ikudome, K.; Saito, I.; Wang, A.H.J. Interactions of deglycosylated cobalt(III)–pepleomycin (green form) with DNA based on NMR structural studies. *Biochemistry* **1997**, *36*, 9995–10005.
69. Hoehn, S.T.; Junker, H.D.; Bunt, R.C.; Turner, C.J.; Stubbe, J. Solution structure of
    Co(III)-bleomycin- OOH bound to a phosphoglycolate lesion containing oligonucleotide: Implications for bleomycin- induced double-strand DNA cleavage. *Biochemistry* **2001**, *40*, 5894–5905.
70. Lui, S.M.; Vanderwall, D.E.; Wu, W.; Tang, X.-J.; Turner, C.J.; Kozarich J.W.; Stubbe, J. Structural characterization of Co**^.^**bleomycin A2 brown: Free and bound to d(CCAGGCCTGG).
    *J. Am. Chem. Soc.* **1997**, *119*, 9603–9613.
71. Manderville, R.A.; Ellena, J.F.; Hecht, S.M. Interaction of Zn(II)·bleomycin with d(CGCTAGCG)(2)-a binding model-based on NMR experiments and restrained molecular-dynamics calculations. *J. Am. Chem. Soc.* **1995**, *117*, 7891–7903.
72. Vanderwall, D.E.; Lui, S.M.; Turner, C.J.; Kozarich, J.W.; Stubbe, J. A model of the structure of HOO-Co.bleomycin bound to d(CCAGTACTGG): Recognition of the d(GpT) site and implications for double-stranded DNA cleavage. *Chem. Biol.* **1997**, *4*, 373–387.
73. Wu, W.; Vaderwall, D.E.; Stubbe, J.; Kozarich, J.W.; Turner, C.J. Interaction of Co**^.^**bleomycin A2 (green) with d(CCAGGCCTGG)_2_: Evidence for intercalation using 2D NMR. *J. Am. Chem. Soc.* **1994**, *116*, 10843–10844.
74. Wu, W.; Vanderwall, D.E.; Turner, C.J.; Kozarich, J.W.; Stubbe, J. Solution structure of Co·bleomycin A2 green complexed with d(CCAGGCCTGG)*.* *J. Am. Chem. Soc.* **1996**, *118*, 1281–1294.
75. Wu, W.; Vanderwall, D.E.; Teramoto, S.; Lui, S.M.; Hoehn, S.T.; Tang, X.-J.; Turner, C.J.; Boger, D.L.; Kozarich, J.W.; Stubbe, J. NMR studies of Co**_._**deglycobleomycin A2 green and its complexes with d(CCAGGCCTGG). *J. Am. Chem. Soc.* **1998**, *120*, 2239–2250.
76. Wu, W.; Vanderwall, D.E.; Turner, C.J.; Hoehn, S.; Chen, J.Y.; Kozarich, J.W.; Stubbe, J. Solution structure of the hydroperoxide of Co(III) phleomycin complexed with d(CCAGGCCTGG)2: Evidence for binding by partial intercalation*. Nucleic Acids Res.* **2002**, *30*, 4881–4891.
77. Zhao, C.Q.; Xia, C.W.; Mao, Q.K.; Forsterling, H.; DeRose, E.; Antholine, W.E.;
    Subczynski, W.K.; Petering, D.H. Structures of HO_2_-Co(III)bleomycin A2 bound to d(GAGCTC)2 and d(GGAAGCTTCC)2: Structure-reactivity relationships of Co and Fe bleomycins. *J. Inorg. Biochem.* **2002**, *91*, 259–268.
78. Povirk, L.F.; Hogan, M.; Dattagupta, N. Binding of bleomycin to DNA: Intercalation of the bithiazole rings*.* *Biochemistry* **1979**, *18*, 96–101.
79. Urata, H.; Ueda, Y.; Usami, Y.; Akagi, M. Enantiospecific recognition of DNA by bleomycin.
    *J. Am. Chem. Soc.* **1993**, *115*, 7135–7138.
80. Glickson, J.D.; Pillai, R.P.; Sakai, T.T. Proton NMR studies of the Zn(II)-bleomycin-A2-poly
    (dA-dT) ternary complex. *Proc. Natl. Acad. Sci. USA* **1981**, *78*, 2967–2971.
81. Chen, D.M.; Sakai, T.T.; Glickson, J.D.; Patel, D.J. Bleomycin-A2 complexes with
    poly(dA-dT)-proton nuclear magnetic-resonance study of the non-exchangeable hydrogens. *Biochem. Biophys. Res. Commun.* **1980**, *92*, 197–205.
82. Booth, T.E.; Sakai, T.T.; Glickson, J.D. Interaction of bleomycin-A2 with poly(deoxyadenylylthymidylic acid)-a proton nuclear magnetic-resonance study of the influence of temperature, pH, and ionic-strength. *Biochemistry* **1983**, *22*, 4211–4217.
83. Sakai, T.T.; Riordan, J.M.; Glickson, J.D. Bleomycin interactions with DNA-studies on the role of the c-terminal cationic group of bleomycin-A2 in association with and degradation of DNA. *Biochim. Biophys. Acta* **1983**, *758*, 176–180.
84. Chien, M.; Grollman, A.P.; Horwitz, S.B. Bleomycin-DNA interactions: Fluorescence and proton magnetic resonance studies. *Biochemistry* **1977**, *16*, 2641–2647.
85. Hiroaki, H.; Nakayama, T.; Ikehara, M.; Uesugi, S. Interaction of bleomycin with deoxyribonucleic-acid oligomer-proton nuclear-magnetic-resonance titration study using novel bleomycin complexes with Ni-2+ and VO-3+. *Chem. Pharm. Bull.* **1991**, *39*, 2780–2786.
86. Sugiura, Y.; Suzuki, T. Nucleotide sequence specificity of DNA cleavage by iron-bleomycin.
    *J. Biol. Chem.* **1982**, *257*, 10544–10546.
87. Yamamoto, K.; Kawanishi, S. Enhancement and alteration of bleomycin-catalyzed site-specific DNA cleavage by distamycin A and some minor groove binders. *Biochem. Biophys. Res. Commun.* **1992**, *183*, 292–299.
88. Suzuki, T.; Kuwahara, J.; Sugiura, Y. Nucleotide sequence cleavage of guanine-modified and DNA with aflatoxin B, dimethyl sulfate, and mitomycin C by bleomycin and deoxyribonuclease *Biochem. Biophys. Res. Commun.* **1983**, *117*, 916–922.
89. Kemsely, J.N.; Loeb, K.; Chow, M.S.; Decker, A.; Shishova, E.Y.; Wasinger, E.C.; Hedman, B.; Hodgson, K.O.; Solomon, E.I. Spectroscopic studies of the interaction of ferrous bleomycin with DNA. *J. Am. Chem. Soc.* **2003**, *125*, 10810–10821.
90. Gobbi, P.G.; Federico, M. What has happened to VBM (vinblastine, bleomycin, and methotrexate) chemotherapy for early-stage Hodgkin lymphoma? *CRC Cr. Rev. Oncol-Hem.* **2012**, *82*, 18–24.
91. Raisfeld, I.H. Pulmonary toxicity of bleomycin analogs. *Toxicol. Appl. Pharm.* **1980**, *56*, 326–336.
92. Raisfeld, I.H.; Chovan, J.P.; Frost, S. Bleomycin pulmonary toxicity-production of fibrosis by bithiazole-terminal amine and terminal amine moieties of bleomycin-A2. *Life Sci.* **1982**, *30*, 1391–1398.
93. Raisfeld, I.H.; Chu, P.; Hart, N.K.; Lane, A. A comparison of the pulmonary toxicity produced by metal-free and copper-complexed analogs of bleomycin and phleomycin*.* *Toxicol. Appl. Pharm.* **1982**, *63*, 351–362.

© 2013 by the authors; licensee MDPI, Basel, Switzerland. This article is an open access article distributed under the terms and conditions of the Creative Commons Attribution license (http://creativecommons.org/licenses/by/3.0/).
